# Spermidine Supplementation Reduces Genetic Damage in the Liver and Bone Marrow of Rodents

**DOI:** 10.1021/acsomega.5c11099

**Published:** 2026-03-10

**Authors:** Janine Barcelos Chacon, Maria Clara Duarte, Michele Oliveira Carvalho, Patrícia Felix Ávila, Isabella Caroline Menon, Giulia de Mello Franco, Bruno Martins Dala Paula, Pollyanna Francielli de Oliveira

**Affiliations:** † Programa de Pós-graduação em Nutrição e Longevidade, 74347 Universidade Federal de Alfenas Alfenas, UNIFAL, Rua Gabriel Monteiro da Silva, 700, Centro, Alfenas, Minas Gerais 37130-001, Brasil; ‡ Laboratório de Genética Humana, Instituto de Ciências da Natureza, Universidade Federal de Alfenas—UNIFAL, Rua Gabriel Monteiro da Silva, 700, Centro, Alfenas, Minas Gerais 37130-001, Brasil; § Laboratório de Nutrição Experimental, Faculdade de Nutrição, Universidade Federal de Alfenas—UNIFAL, Rua Gabriel Monteiro da Silva, 700, Centro, Alfenas, Minas Gerais 37130-001, Brasil

## Abstract

Mutations in DNA constitute the initial step in the process of carcinogenesis. While diets high in sugar increase the risk of various diseases, a balanced diet rich in bioactive compounds can mitigate DNA damage. Spermidine (SPD) is a polyamine with reported effects on autophagy induction, inflammation reduction, and the enhancement of immune function. Within this context, we investigated the effects of SPD supplementation [10 and 30 mg/kg body weight (bw)] in male Swiss mice over a period of 44 days. The animals were divided into two groups: one fed a standard diet (SD—Nuvilab CR1), and the other fed a standard diet enriched with 30% sucrose (SU) (SDSU). On day 44, the mutagenic agent methylmethanesulfonate [MMS 40 mg/kg bw intraperitoneal (ip)] was administered to induce mutagenicity. On day 45, bone marrow and liver cell samples were collected to evaluate the chemopreventive potential and oxidative stress. The results showed that SDSU significantly reduced feed intake and increased water consumption. None of the treatments exhibited cytotoxic or mutagenic effects. Animals treated with SPD 30 mg/kg bw combined with MMS, under both SD and SDSU conditions, showed a reduction in the frequency of micronucleated polychromatic erythrocytes (MNPCEs) in bone marrow and reduced DNA damage in liver cells, particularly when they were not associated with SU consumption. Additionally, significantly increased catalase (CAT) and superoxide dismutase (SOD) levels were observed, suggesting that the genoprotective effect against induced damage may be at least partially related to mobilization of the endogenous antioxidant defense system.

## Graphical abstract

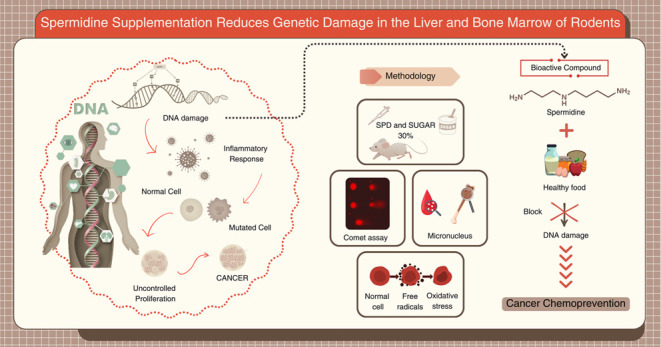

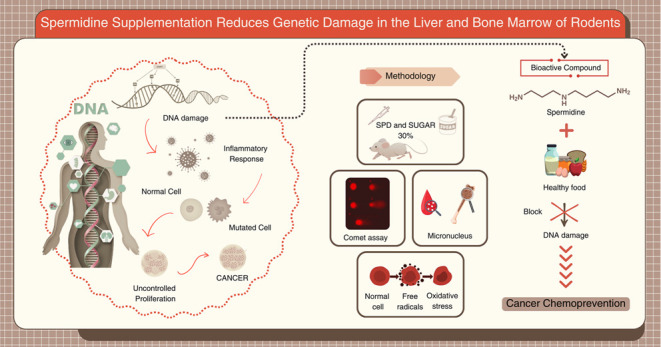

## 1 Introduction

Unbalanced diets, rich in ultraprocessed foods, have been consistently associated with metabolic dysregulation and an increased risk of cancer, partly due to their ability to promote oxidative stress and genomic instability.^1^ On the other hand, a high consumption of sucrose (SU), which is very common in a diet high in processed foods, increases glucose metabolism levels for insulin secretion, which can trigger the formation of reactive oxygen species (ROS) through the mitochondrial respiratory chain.^2^ Excessive sugar intake enhances hepatic lipogenesis and mitochondrial overload, favoring the generation of ROS and consequent DNA damage, particularly in metabolically active tissues, such as the liver. These alterations may compromise genomic integrity and contribute to carcinogenic processes if not efficiently counteracted.^3^ Endogenous antioxidant defense systems, including superoxide dismutase (SOD) and catalase (CAT), play a central role in mitigating oxidative damage by detoxifying ROS and preserving cellular homeostasis.^4^ Therefore, preventing the occurrence of such errors may promote an integrated and homeostatic cellular response, limiting the emergence of mutations and representing an important strategy for reducing the incidence of different types of cancer.^5^

Bioactive compounds may represent an important therapeutic approach for reducing DNA damage. Many of these compounds can act as chemopreventive agents when used alone or in combination with other substances and/or chemotherapeutic agents, as well as with dietary strategies aimed at interfering with the process of oncogenesis.^6^ Polyamines are an example of such bioactive compounds and play an important role in protein and nucleic acids synthesis and stability, cell proliferation, differentiation, apoptosis, and the regulation of oxidative stress.^7^

Spermidine (SPD) is a polyamine compound (C7H19N3) found in ribosomes and living tissues, where it performs several metabolic functions.^8–10^ Mice supplemented with SPD [110 mg/kg body weight (bw)] for 6 months exhibited reduced telomeric attrition in cardiac tissue and attenuation of necrotic changes in the liver compared to nonsupplemented animals.^11^ Additionally, SPD has been reported to inhibit transplantable tumor growth, stimulate immune surveillance when combined with chemotherapeutic agents, and contribute to the maintenance of cellular homeostasis.^5,10^

Although SPD exhibits promising activity, comprehensive investigations regarding dosage, prolonged exposure time, lesion type, and mechanism of action remain limited, hindering a clear understanding of its direct effects on genetic material. Moreover, to date, no studies have explored the effects of SPD when combined with a high-sugar diet. Within this context, this study investigated the effects of SPD supplementation in rodents fed a standard diet (SD) high in added SU, both before and after the induction of DNA damage.

Methylmethanesulfonate (MMS) is an organic compound with toxicity that is based on the transfer of methyl groups to DNA, which leads to the formation of mutagenic lesions. This property makes it a valuable tool for inducing mutations in laboratory settings, allowing researchers to study gene function and DNA repair mechanisms. Within this experimental framework, the evaluation of compounds capable of modulating MMS-induced genetic damage becomes particularly relevant.^12^

## 2 Materials and Methods

### 2.1 Chemicals

SPD, obtained from Sigma-Aldrich (CAS: 124-20-9), was diluted in distilled water immediately before use at doses of 10 and 30 mg/kg of body weight (bw), established based on literature in pilot trials,^13,14^ and administered orally (po) to the animals. For DNA damage induction, the mutagenic agent MMS (Sigma-Aldrich, CAS: 66-27-3) was used as a positive control (PC) at a dose of 40 mg/kg bw, diluted in distilled water and administered intraperitoneally (ip).^15^

### 2.2 Determination of Antioxidant Potential, Total Phenolic Content, and Total Flavonoid Content

The extract was prepared according to Oliveira et al.^16^ with modifications. Briefly, 1 g of SD sample, SPD standard, or SU was added to 15 mL of an 80% ethanol solution (v/v), vortexed on a benchtop mixer for 1 min, and then sonicated at 40 kHz for 20 min at 25 °C, vortexed for 1 min more, and centrifuged at 4,000*g* for 10 min at 4 °C (Fanem Excelsa Baby Centrifuge Model 206-BL, Guarulhos, São Paulo, Brazil). The supernatant was collected, and the extracts were stored frozen at −18 °C until analysis.

The antioxidant potential was determined using spectrophotometric methods based on the decolorization of the stable nitrogen radical 2,2-diphenyl-1-picrylhydrazyl (DPPH), achieved through neutralization by antioxidant compounds present in the sample.^16^ The results were expressed as mmol of TROLOX equivalents per gram of sample (mmol of TE/g). To determine total phenolic content (TPC) and total flavonoid content (TFC), the methodology described by Ávila et al.^17^ was used, with results expressed as mg gallic acid equivalents (mg GAE/g) and mg catechin equivalents per gram of sample (mg CE/g), respectively.

### 2.3 Animals

*Mus musculus* male mice, 4–5 weeks old (33 g), were purchased from the Bioterism Center of the Federal University of Alfenas (UNIFAL-MG) and were housed in cages under 23 ± 2 °C and 50 ± 5% relative humidity conditions with a 12 h/12 h light and dark cycle. The mice had free access to drinking water and a SD (irradiated Nuvilab CR-1, Supporting Information, S1). All experimental procedures involving animals were approved by the Ethics Committee on the Use of Animals—CEUA of UNIFAL-MG, Brazil (0040/2022).

### 2.4 Experimental Protocol

The experimental period lasted for a total of 52 days (Figure 1). Animals were acclimated for 1 week, and treatments were initiated on day 8 and maintained for 45 days. In addition to the negative control (NC) and PC (MMS 40 mg/kg bw) groups, animals were divided into 2 main experimental groups: one received a SD supplemented with SPD (10 and 30 mg/kg bw) and another received a SD enriched with 30% SU (SDSU), added to the drinking water, and/or supplemented with SPD (10 and 30 mg/kg bw). Peripheral blood samples were collected for mutagenicity assessment 48 h and 44 days after the initiation of treatments (10th and 51st days of start experimentation, respectively). On day 44 of treatment (51st day of experimentation), following blood collection, the mutagenic agent MMS (40 mg/kg bw, ip) was administered to all groups (except for the NC group) to induce DNA lesions. Bone marrow and liver samples were collected 24 h later (on the 45th day of treatment, the 52nd day of experimentation) for the evaluation of chemopreventive potential and oxidative stress, following the euthanasia of animals with ketamine (300 mg/kg bw) and xylazine (30 mg/kg bw), administered ip. Food and water intake was monitored daily, and blood glucose levels were quantified before the initiation of treatment and on the 44th day. Animals were not fasted overnight or at any time prior to anesthesia or terminal blood and tissue collection. A short fasting period of 4 h was applied exclusively for blood glucose measurements before the initiation of treatment and on the 44th day.

**Figure 1:**
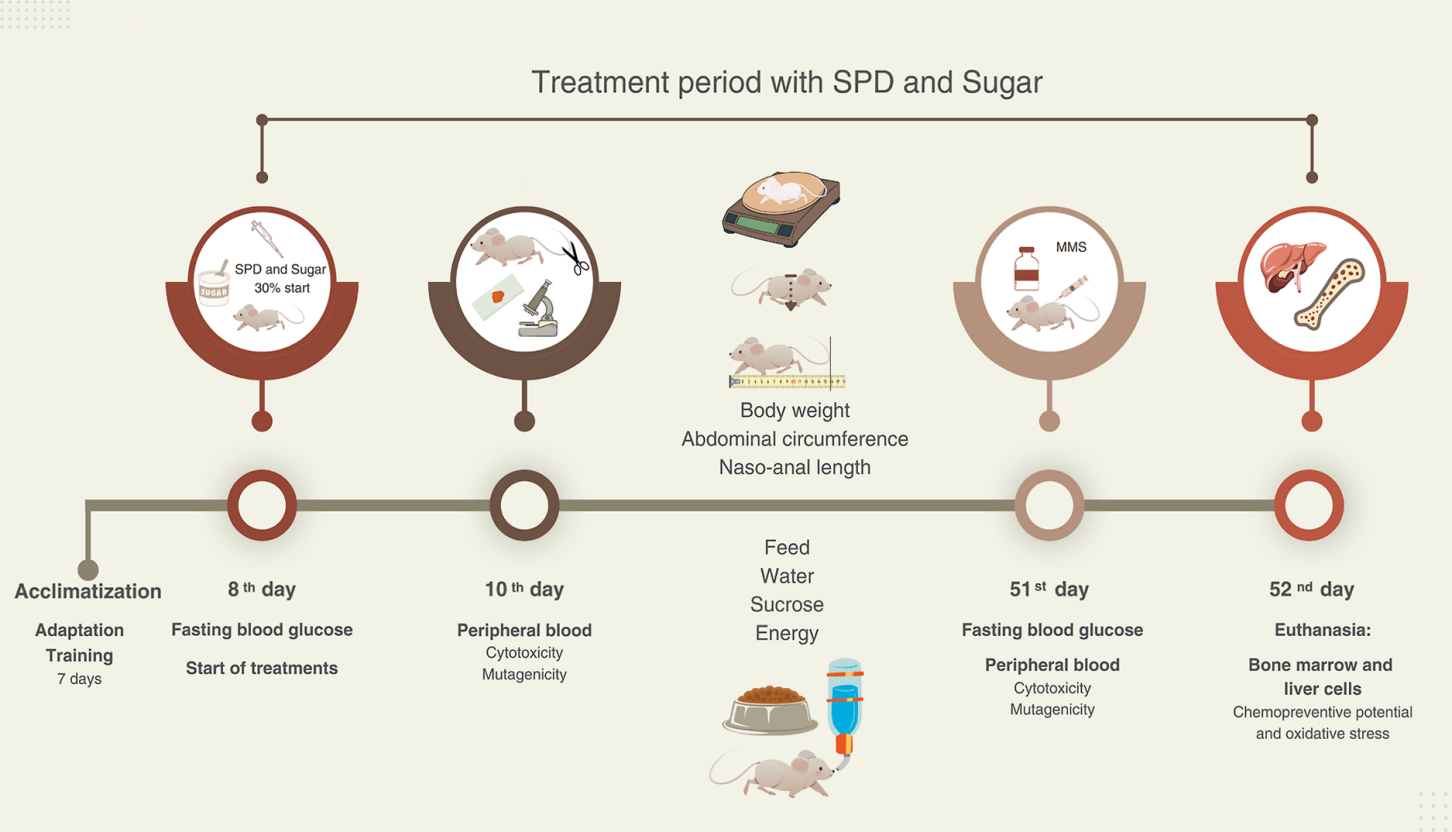
Experimental protocol timeline. SPD—SPD 30—spermidine 30 mg/kg body weight (bw). MMS—methylmethanesulfonate 40 mg/kg bw. During the acclimatization period, animals were fed only standard diet (SD—Irradiated Nuvilab CR1). On the 1st day of treatment, blood glucose was quantified after 4 h of fasting, followed by the initiation of SU (30%) supplementation in the drinking water and oral administration of SPD, which continued until the end of the experimental period (euthanasia). After a new quantification of fasting blood glucose (with 4 h of fasting), on day 51, with the animals were kept under treatment, MMS was administered a DNA damage-inducing agent. After 24 h of MMS administration (day 52), animals were euthanized for sample collection. Source: from the author.

Throughout the experimental period, the mass (measured using a scale, Marte BL3200H 3200 g), the naso-anal length (NAL), and abdominal circumference (AC) (measured with a tape measure) were obtained in order to determine the Lee index according to the following formula

$$[\sqrt[{3}]{body\;weight\;(g)}\;/NAL\;\left(cm\right)]$$

The assessment of the weight of different organs (heart, lungs, kidneys, spleen, gastrointestinal tract, and carcass) was performed at the time of euthanasia using a precision scale (Marte BL3200H). The results were expressed as relative weight ([organ weight/body weight] × 100).^18^

### 2.5 Micronucleus Test

The micronucleus (MN) test was performed on peripheral blood and bone marrow samples of Swiss mice. A drop of peripheral blood obtained through a small incision at the tip of the tail was applied directly onto the surface of a clean, dry slide, followed by smearing, drying fixation, and staining with Giemsa.^15,19^

Bone marrow cells were obtained by flushing the femurs with fetal bovine serum with a syringe. The cell suspension was centrifuged at 800 rpm for 5 min, and smears were prepared from the pellets on glass slides. After air-drying, slides were fixed and stained according to the protocols described by Furtado et al. and Schmid et al.^20,21^

Analyses were conducted under an optical microscope at a total magnification of 1000×. The frequency of micronucleated polychromatic erythrocytes (MNPCEs) was determined by counting 4000 polychromatic erythrocytes (PCEs) per animal. Cytotoxicity was assessed using the same slides prepared for the MN test, by counting 2000 total erythrocytes per animal and quantifying those that were classified as normochromatic (NCEs) and PCEs to calculate PCE/NCE + PCE ratio.^22^

### 2.6 Comet Assay

Liver tissue was mechanically disaggregated, and the resulting cell suspension was mixed with low-melting-point agarose and spread onto microscope slides precoated with normal-melting-point agarose. Slides were then immersed in lysis solution and stored at 4 °C for 24 h. DNA migration was performed by electrophoresis (25 V, 300 mA, 7 W) in alkaline buffer (pH > 13.4, 4 °C, dark) for 20 min. After electrophoresis, slides were neutralized, fixed in absolute ethanol, and stained immediately before analysis with GelRed Nucleic Acid Stain (1:1100).^23,24^ Images of 100 nucleoids per animal were captured using a fluorescence microscope (Olympus, Tokyo, Japan) at 40× magnification and a TRITC filter and analyzed with ImageJ software (v1.3.1, OpenComet plugin), using the Tail DNA % as a comparative parameter.

### 2.7 Endogenous Enzymatic Antioxidant System: Superoxide Dismutase and Catalase Activities

Liver homogenates were prepared from approximately 100 mg of tissue in 1 mL of 50 mmol/L phosphate saline buffer (PSB, pH 7.0). Protein concentration was determined using the Bradford method.^25^

For SOD activity determination, the liver homogenate (200 μL) containing pyrogallol–15 mM (100 μL), MTT—1.25 mM (40 μL), and PSB-pH 7.0 (660 μL) was incubated for 5 min at 37 °C. The reaction was stopped by adding dimethyl sulfoxide (DMSO, 1000 μL), and the absorbance was measured at 570 nm using a spectrophotometer. Each SOD unit (U) corresponded to 50% pyrogallol oxidation. For the final quantification, blank sample values were subtracted and divided by the standard. Results were expressed as U/mg of protein.^26^

The CAT activity was determined based on the rate of hydrogen peroxide (H_2_O_2_) decomposition at 240 nm. The reaction mixture, containing H_2_O_2_ (10 mM) (980 μL) and liver homogenate diluted in PSB (PBS 20 μL), was incubated at 25 °C for 1 min. Absorbance readings were taken between 1 and 3 min, and the results were expressed in U/g protein using the molar absorptivity coefficient of H_2_O_2_ at 240 nm = 39.4 M–1 cm^–1^ H_2_O_2_ concentration calculation.^27^

### 2.8 Statistical Analysis

Statistical analyses were performed with GraphPad Prism 8.4.3 (GraphPad Software, San Diego, CA, USA). One-way analysis of variance (ANOVA) was used to assess differences. Results are presented as the mean ± standard deviation, and a *p* value <0.05 was considered statistically significant. In cases where *p* < 0.05, treatment means were compared with each other using Tukey’s method.

## 3 Results and Discussion

### 3.1 Antioxidant Potential, TPC, and TFC

The results of the antioxidant potential, TPC, and TFC in SD, SU, and SPD samples are presented in Table 1. The antioxidant potential of SU and SPD, as assessed by the DPPH method, was approximately 31 and 213 times lower than that observed in the SD. This finding indicates a minimal contribution of these components to the total antioxidant potential of the diet. SU and SPD exhibited undetectable levels of TFC and TPC, whereas the diet contained 0.60 mg EC/g and 1.54 mg EAG/g, respectively.

**Table 1:** Antioxidant Potential, TFT, and TPT Present in SD, SU, and SPD

sample	^d^DPPH (nmol ET/g)	TFC (^c^mg EC/g)	TPC (^b^mg EAG/g)
SD^a^	3240 ± 90	0.60 ± 0.03	1.54 ± 0.03
SU	103.64 ± 10.33	-	-
SPD	15.22 ± 0.57	-	-

Results are media ± standard deviation.

a Standard diet Irradiated Nuvilab CR-1.

b Equivalent in gallic acid (mg EAG/g).

c Equivalent in catechin (mg EC/g).

d Equivalents in trolox (nmol ET/g). TPC – Total phenolic content, TFC – Total flavonoid content, SPD – spermidine, SU – sucrose, DPPH - 2,2-diphenyl-1-picryl-hydrazyl.

Approximately 2 × 10^4^ harmful events to DNA occur daily in all cells of the human body. A significant portion of this damage is caused by ROS. The effect of excessive ROS production and/or the imbalance of cellular antioxidant defense systems is known as oxidative stress, which can trigger various types of DNA damage, including oxidized purines and pyrimidines, single-strand breaks, double-strand breaks, and abasic sites. Detecting the damage and its biological targets is essential for elucidating its mechanisms of activity and enabling the development of antioxidant intervention strategies to combat diseases associated with oxidative stress.^27–29^ To minimize the impact of an imbalance between ROS and antioxidants, investigations of substances with antioxidant potential are essential.^30^

Several studies have identified plant-derived compounds as promising sources of natural antioxidants. For example, oregano has been reported as a potent antioxidant source. Using the ABTS assay, they evaluated the antioxidant activity of the essential oil and extracts from Greek oregano and common oregano, finding values between 3.40 × 10^5^ and 3.97 × 10^5^ nmol Trolox/g, with extracts showing higher antioxidant potential.^31^ Camu–camu (*Myrciaria dubia*) is one of the richest natural sources of vitamin C (1150 mg/100 g). The fruit exhibited high antioxidant activity, with values of 5763 × 10^–6^ nmol/g (DPPH) and 6981 × 10^–6^ nmol/g (ABTS), surpassing those found for SD, SU, and especially SPD in our study.^32^ Xie et al. analyzed germinated barley seedlings treated with compounds that enhance SPD degradation. Seedlings treated with 2.5 mM aminoguanidine hydrochloride showed a DPPH value of 14,520 ± 540 nmol TE/g, indicating that while SPD has some antioxidant activity, it is lower than that of compounds like quercetin.^33^ The latter, whose chemical structure facilitates hydrogen donation and free radical stabilization, presented an IC_50_ of 26.94 nmol/g.^34^

### 3.2 Food, Water, SU, and Total Energy Intake

Food, water, and SU intake, as well as total energy intake, were monitored throughout the experimental period (Figure 2A–D). The groups consuming SU (SDSU, SDSU + SPD 10, and SDSU + SPD 30) showed significantly lower food intake compared to the groups not receiving SU (SD, SD + MMS, SD + SPD 10, and SD + SPD 30). The SDSU + SPD 30 group was not significantly different from the SDSU group (Figure 2A). Regarding water intake, the SDSU + SPD 10 group exhibited significantly higher water consumption compared to those of the SD, SD + MMS, SD + SPD 10, and SD + SPD 30 groups. The SDSU and SDSU + SPD 30 groups also showed significantly higher water intake compared to the SD + MMS, SD + SPD 10, and SD + SPD 30 groups (Figure 2B). SU intake (Figure 2C) and total energy intake (Figure 2D) showed no significant differences among the groups.

**Figure 2:**
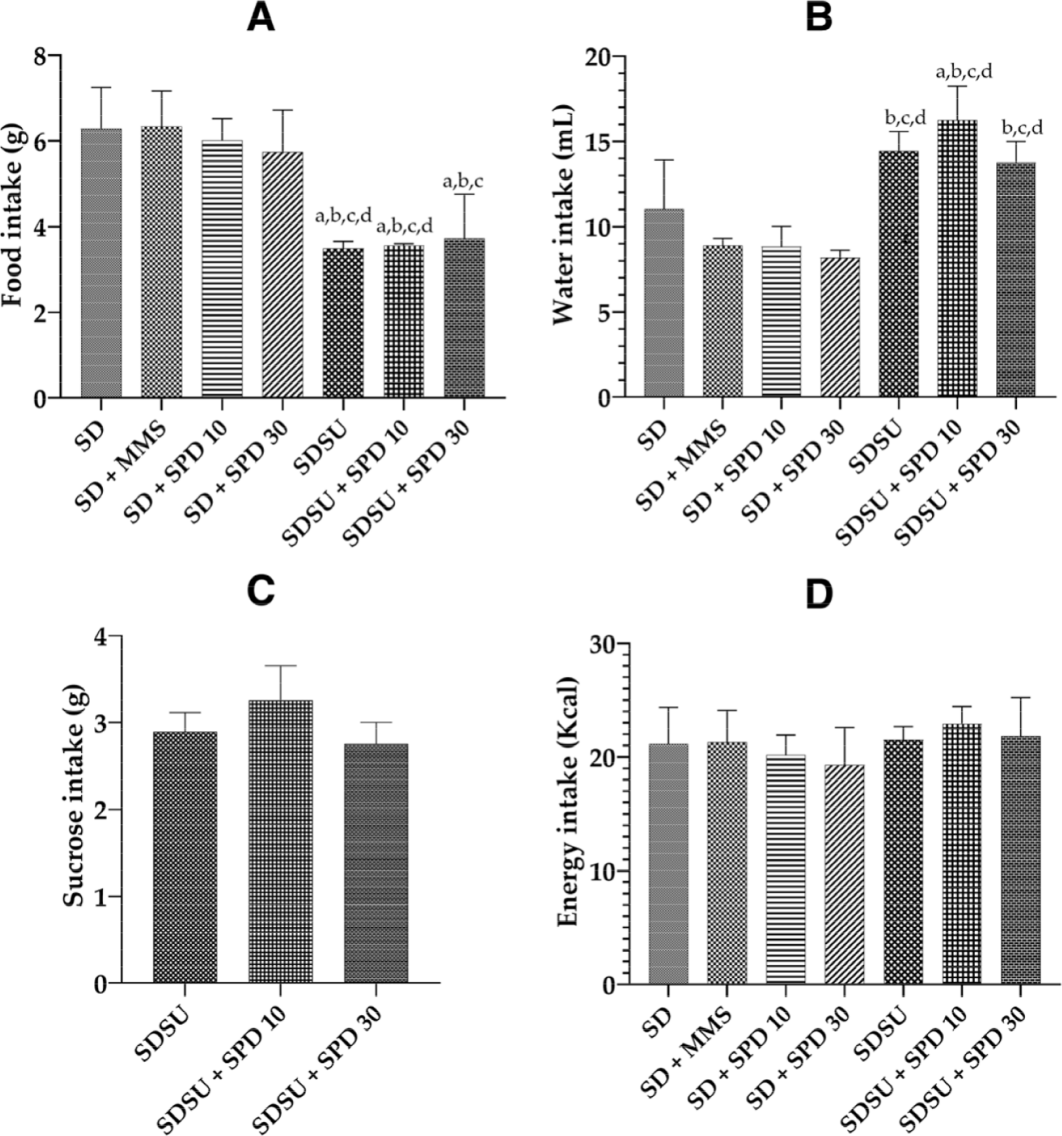
(A) Food (g), (B) water (mL), (C) SU (g), and (D) energy intake (kcal) observed during the experimental period. SD—standard diet Irradiated Nuvilab CR-1 (NC), MMS—methylmethanesulfonate 40 mg/kg body weight (bw) (PC), SU—sucrose, SPD 10—spermidine 10 mg/kg bw, and SPD 30—spermidine 30 mg/kg bw. Values are mean ± standard deviation of daily consumption. Significance level for *p* < 0.05 (one-way ANOVA and Tukey’s test, average *n* = 9 animals/treatment group). aSignificantly different from the SD group, bsignificantly different from the SD + MMS group, csignificantly different from the SD + SPD 10 group, and dsignificantly different from the SD + SPD 30 group.

The reduction in food intake observed in animals consuming SU may be explained by the complex regulation of energy homeostasis mediated by interactions among hormones and neuromodulators in the hypothalamus. Chemerin, an adipokine encoded by the retinoic acid receptor responder 2 (Rarres2) gene, plays a key role in regulating appetite and body weight and becomes active when energy demands are met.^35^ Thus, SU consumption, as a rapid energy source, may activate chemerin signaling, leading to reduced food intake, even over short periods, as a compensatory mechanism to balance energy intake. In agreement with our findings, genetically modified mice with free access to different SU concentrations showed reduced food intake across all genotypes evaluated.^36^ Similarly, Korgan et al. reported that mice receiving 10% SU in drinking water exhibited lower food intake compared with mice provided only standard chow and water.^37^

The increased water intake observed in all groups consuming SU can be explained by its palatability, which promotes increased water consumption.^38^ In agreement with our findings, Wu et al. demonstrated that mice treated with 60 mg/mL of SU drinking water consumed significantly more water than control animals.^39^

### 3.3 Murinometric Assessments

Murinometric parameters, including body mass, AC, and NAL, were measured throughout the experimental period for calculating the Lee index (Figure 3A–D). Overall, animals gained an average of 7.9 g over the entire experimental period, with no significant differences were observed between groups in terms of body mass gain (Figure 3A), AC (Figure 3B), or NAL (Figure 3C). Initial AC showed an average of 8.0 ± 0.5 cm, while final AC was 8.4 ± 0.5 cm, resulting in an average gain of 0.4 ± 0.5 cm (Figure 3B). Initial NAL showed an average of 9.8 ± 0.5 cm, while final NAL was 10.5 ± 0.4 cm, resulting in an average increase of 0.7 ± 0.6 cm (Figure 3C).

**Figure 3:**
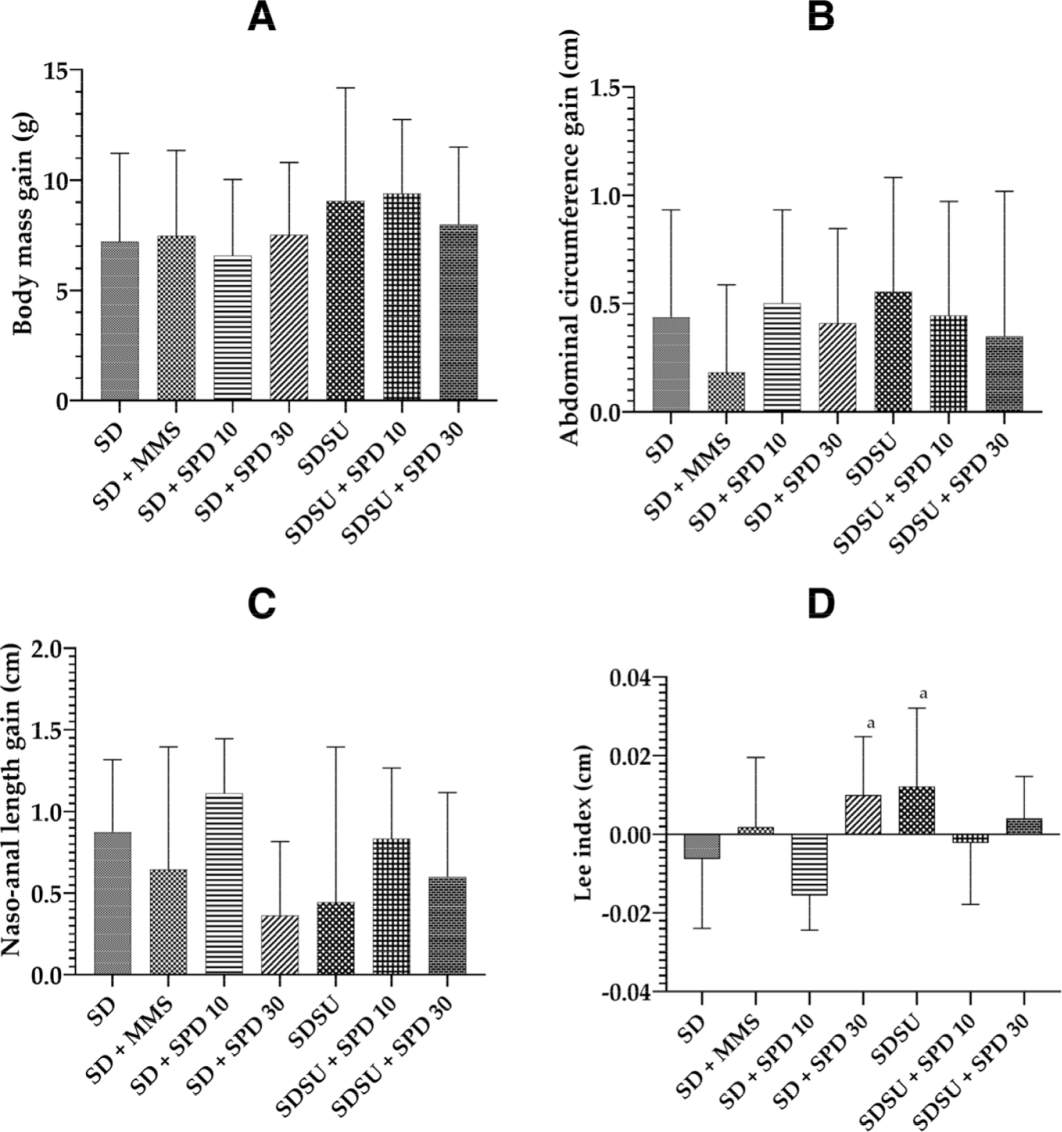
(A) Body mass (cm), (B) AC (cm), (C) NAL gain (cm), and (D) Lee index (cm) of Swiss mice in different treatments. AC—abdominal circumference, NAL—naso-anal length, SD—standard diet Irradiated Nuvilab CR-1 (NC), MMS—methylmethanesulfonate 40 mg/kg body weight (bw) (PC), SU—sucrose, SPD 10—spermidine 10 mg/kg bw, and SPD 30—spermidine 30 mg/kg bw. Values are mean ± standard deviation. Significance level for *p* < 0.05 (one-way ANOVA and Tukey’s test, average *n* = 9 animals per group). aSignificantly different from the SD + SPD 10 group. AC gain represents the difference in circumference between the first (8th) and last (51st) day of treatment.

The analysis of the Lee index (calculated as the difference between final and initial values) showed that the SD + SPD 30 and SDSU groups exhibited significantly higher Lee index values compared to the SD + SPD 10 group (Figure 3D). To evaluate whether SU and/or SPD consumption influenced blood glucose levels, this parameter was assessed at baseline (0 h) and after 44 days of treatment (51st day of experimentation). These results are presented in Supporting Information (S2). At the initial evaluation (0 h), all animals were still being fed with SD (Irradiated Nuvilab CR1), and no significant differences were observed between the groups (S2 A–C). Organ weights, including the heart, lungs, kidneys, spleen, gastrointestinal tract, and carcass, were assessed to identify potential associations with body weight changes. No significant differences were observed in the relative weights of any organs among the experimental groups (S3 A–F).

Regarding murinometric parameters, previous studies have indicated that SU consumption does not affect the body weight of mice or lead to overweight.^37,40^ Likewise, studies involving SPD supplementation have demonstrated that treatment with this polyamine does not result in significant differences in body weight gain.^11,41^

AC is an important parameter for assessing abdominal adiposity and refining body mass index (BMI), which is a nonspecific index of adipose tissue in that region.^42^ Similarly to this study, Angéloco et al. found no significant differences in AC gain between the group of Wistar rats treated with a high SU diet for 4 weeks and the NC group.^43^ Conversely, Wistar rats fed a high SU diet for 6 months exhibited significant increases in AC,^44^ suggesting that the duration of SU exposure is a variable that may influence this parameter.

In humans, linear growth is influenced by the interaction of multiple genetic variants and environmental factors, with nutrition playing a key regulatory role.^45^ In rodents, the NAL is a relevant indicator of growth and development. Unlike the present study, which found no significant differences in NAL among groups, Alves et al. reported greater NAL gain in obese Wistar rats fed a high-fat, high-SU diet for 7 weeks compared with the NC group.^46^ Similarly, genetically modified rats exposed to a hypercaloric (high-fat) diet for 4 weeks showed significant increases in NAL.^45^ These discrepancies may be attributed to differences in diet composition, particularly the absence of a high-fat component in the present study.

Collectively, AC, weight, and NAL measurements allow for the calculation of the Lee index, a BMI used in rodents. Studies have shown no significant differences in this parameter in Wistar rats supplemented with SU in water compared to the CN group.^47^ However, in rodents fed a high-fat, high-SU diet, an increase in this index was observed,^46^ reinforcing the importance of dietary composition in the modulation of this parameter.

### 3.4 Mutagenic and Antimutagenic Potential

The mutagenicity of the treatments was investigated in peripheral blood (Figure 4E) at 48 h and 44 days (Figure 4C,D), alongside the evaluation of the PCE/NCE + PCE ratio (Figure 4A,B). No statistically significant differences were observed in the PCE/NCE + PCE ratio or in the MNPCE frequency in any experimental groups compared to the SD group at either collection time (48 h and 44 days), indicating the absence of mutagenicity and cytotoxicity of the treatments (Figure 4A–E).

**Figure 4:**
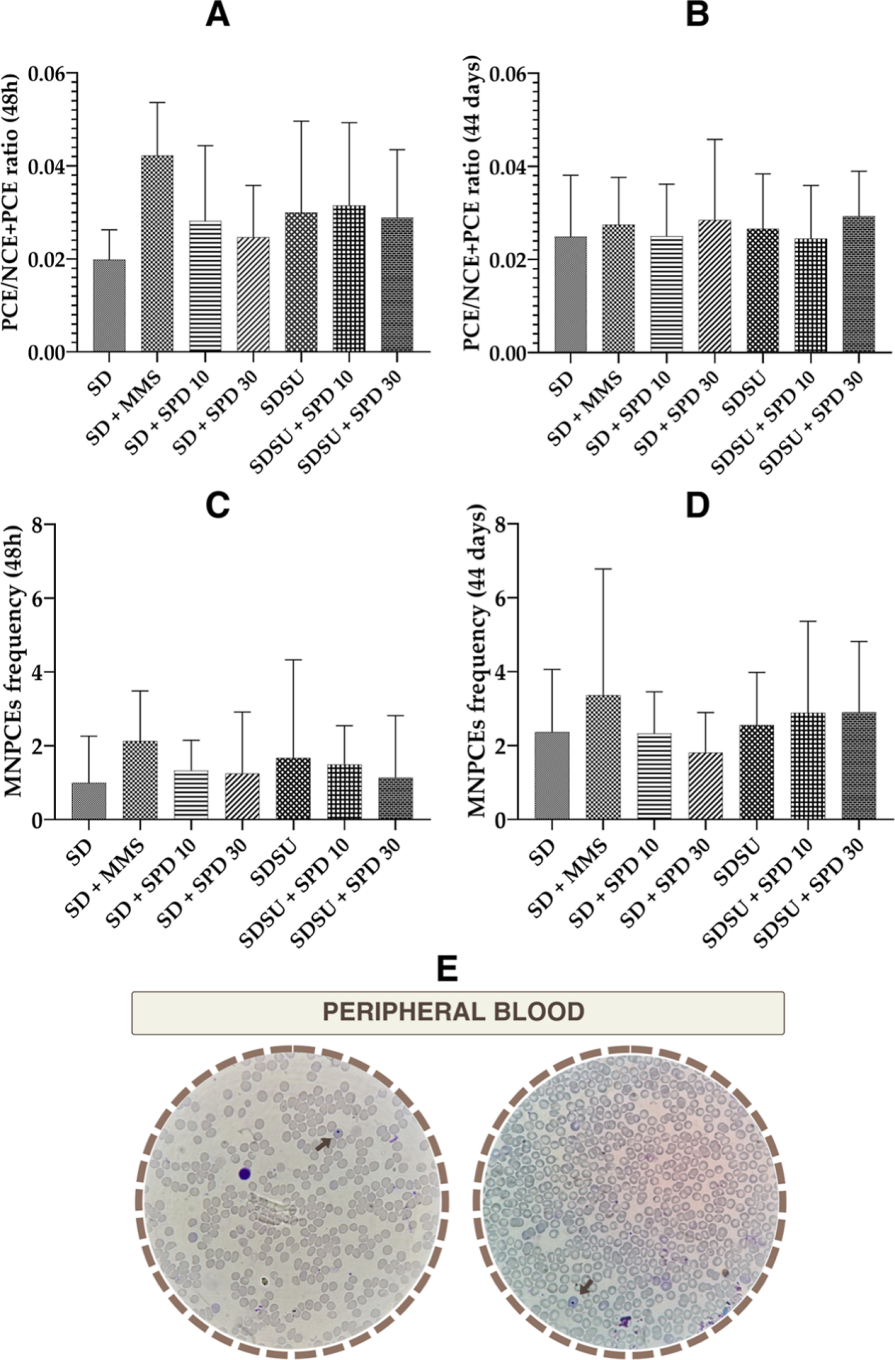
(A) PCE/NCE + PCE ratio at 48 h, (B) PCE/NCE + PCE ratio at 44 days, (C) MNPCE frequency at 48 h, (D) MNPCE frequency at 44 days, and (E) photomicrography of peripheral blood cells of Swiss mice (total increase of 100x). NCE—normochromatic erythrocytes, PCE—polychromatic erythrocytes, MNPCEs—micronucleated polychromatic erythrocytes, SD—standard diet Irradiated Nuvilab CR-1 (NC), MMS—methylmethanesulfonate 40 mg/kg body weight (bw) (PC), SU—sucrose, SPD 10—spermidine 10 mg/kg bw, and SPD 30—spermidine 30 mg/kg bw. Values are mean ± standard deviation. Significance level for *p* < 0.05 (one-way ANOVA and Tukey’s test, average *n* = 6 animals per group). 48 h and 44 days correspond, respectively, to the 2nd day of treatment (10th day and 52nd day of experimentation).

Antimutagenicity was investigated in bone marrow cells (Figure 5A,B) and liver cells (Figure 6A). No statistically significant differences were observed in the PCE/ (PCE + NCE) ratio across any treatment group, indicating the absence of cytotoxicity in bone marrow cells (Figure 5A). All treatment groups showed statistically significant differences compared to the SD group. The SD + SPD 30 + MMS and SDSU + SPD 30 + MMS groups exhibited a significant reduction in the frequency of MNPCEs compared to the SD + MMS group (by 41% and 27%, respectively), indicating a potential genoprotective effect. The SD + SPD 30 + MMS treatment group showed significant differences relative to the SD + SPD 10 + MMS group, while the SDSU + MMS and SDSU + SPD 10 + MMS groups displayed significant differences compared to the SD + SPD 30 + MMS group. Finally, the SDSU + SPD 30 + MMS group demonstrated significant differences when compared with the SD + SPD 10 + MMS group (Figure 5B).

**Figure 5:**
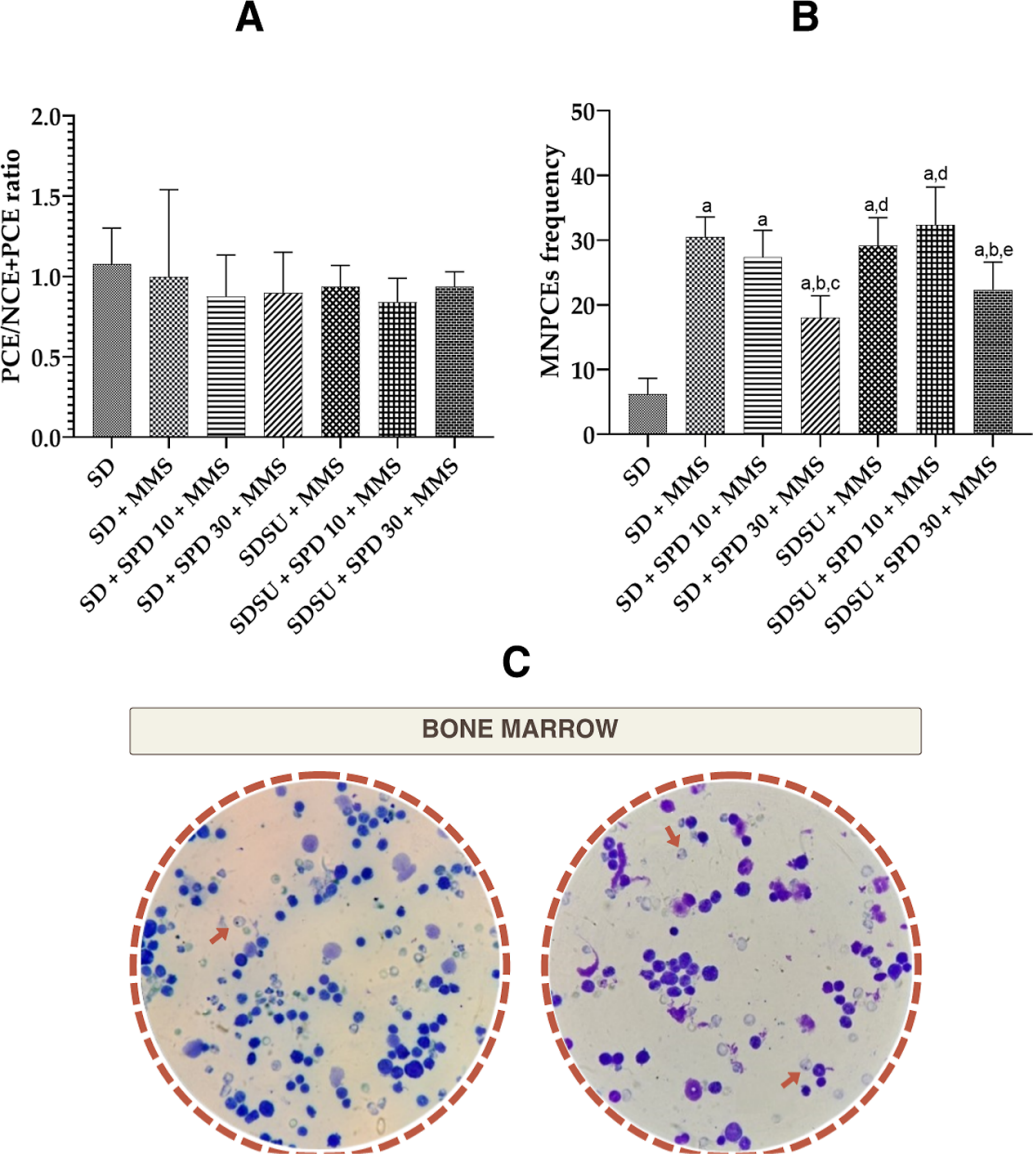
(A) PCE/NCE + PCE ratio, (B) MNPCE frequency, and (C) photomicrography of bone marrow observed after 45 days of the treatment (total increase of 1000×) of Swiss mice. NCE—normochromatic erythrocytes, PCE—polychromatic erythrocytes, MNPCEs—micronucleated polychromatic erythrocytes, SD—SD Irradiated Nuvilab CR-1 (NC), MMS—methylmethanesulfonate 40 mg/kg body weight (bw) (PC), SU—sucrose, SPD 10—spermidine 10 mg/kg bw, SPD 30—spermidine 30 mg/kg bw. Values are mean ± standard deviation. The animals received different treatments for 45 days, and on the 44th day (51st experimental day), the mutagenic agent methylmethanesulfonate (MMS 40 mg/kg bw) was administered. Significance level for *p* < 0.05 (one-way ANOVA and Tukey’s test, average *n* = 6 animals per group). aSignificantly different from the SD group; bsignificantly different from the SD + MMS group; csignificantly different from the SD + SPD 10 + MMS group; dsignificantly different from the SD + SPD 30 + MMS group; and esignificantly different from the SDSU + SPD 10 + MMS group.

**Figure 6:**
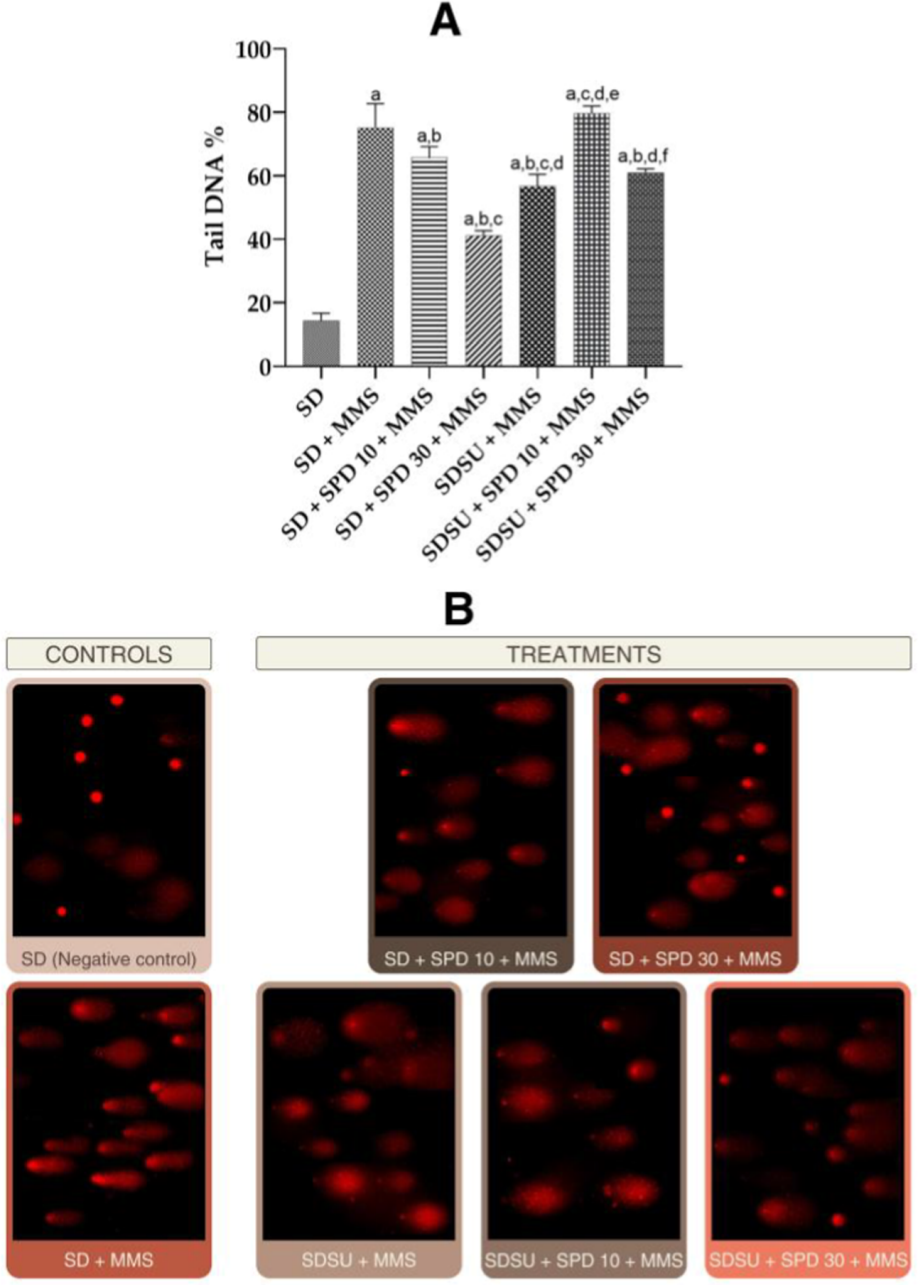
(A) DNA percent in comet tail and (B) photomicrographs of the different treatments performed on liver cells of Swiss mice and their respective controls. SD—standard diet Irradiated Nuvilab CR-1 (NC), MMS—methylmethanesulfonate 40 mg/kg body weight (bw) (PC), SU—sucrose, SPD 10—spermidine 10 mg/kg bw, and SPD 30—spermidine 30 mg/kg bw. Values are mean ± standard deviation. The animals received different treatments for 45 days, and on the 44th day (51st experimental day), the mutagenic agent methylmethanesulfonate (MMS 40 mg/kg bw) was administered. Significance level for *p* < 0.05 (one-way ANOVA and Tukey’s test, average *n* = 6 animals per group). aSignificantly different from the SD group; bsignificantly different from the SD + MMS group; csignificantly different from the SD + SPD 10 + MMS group; dsignificantly different from the SD + SPD 30 + MMS group; esignificantly different from the SDSU + MMS group; and fsignificantly different from the SDSU + SPD 10 + MMS group.

Regarding the effects on liver cells, the comet assay revealed significantly lower DNA damage in the SDSU + MMS (30% reduction), SD + SPD 10 + MMS (15% reduction), SD + SPD 30 + MMS (56% reduction), and SDSU + SPD 30 + MMS (23% reduction) groups compared with the SD + MMS group. The SDSU + SPD 10 + MMS group exhibited a significantly higher percentage of DNA damage than the SD + SPD 10 + MMS group, whereas the SD + SPD 30 + MMS and SDSU + MMS groups showed significant reductions in DNA damage relative to this same comparison. In addition, the SDSU + MMS, SDSU + SPD 10 + MMS, and SDSU + SPD 30 + MMS groups exhibited significantly higher DNA damage levels compared with the SD + SPD 30 + MMS group. The SDSU + SPD 10 + MMS group also showed significantly greater DNA damage than the SDSU + MMS group. Conversely, the SDSU + SPD 30 + MMS group exhibited significantly lower DNA damage, compared with the SDSU + SPD 10 + MMS group. As expected, all treatment groups displayed significantly higher DNA damage percentages than the SD group (Figure 6A,B).

Overall, our findings suggest that supplementation with SPD in rodents did not trigger early signs of toxicity, as it neither reduced water consumption nor altered the average daily energy intake and murinometric parameters. The acute and sub chronic genotoxic safety of SPD supplementation in SD and SDSU was investigated in peripheral blood by monitoring MNPCE frequency, a sensitive biomarker of DNA damage commonly used in mutagenicity research both in vitro and in vivo in different cell types. MNs are formed during the metaphase–anaphase transition of mitosis due to chromosomal breakage (clastogenesis) or malformation of the mitotic spindle apparatus malfunction (aneugenesis), often triggered by high free radical production during oxidative stress. These events can promote the development of chronic diseases, including cancer.^48^ In addition, quantifying PCE/ (PCE + NCE) ratio was assessed to evaluate cytotoxicity. Moreover, considering the direct relationship between mutagenesis and carcinogenesis, and aiming to clarify whether SPD could protect genetic material from harmful events leading to malignant transformation in a SU rich diet, a common dietary pattern today, we evaluated the genoprotective potential of SPD supplementation in bone marrow and liver cells by a comet assay after 45 days of exposure.

The results demonstrate the absence of both acute and chronic cytotoxic and mutagenic effects, while a genoprotective effect was observed in the peripheral blood of animals fed SD and SDSU diets and supplemented with 30 mg/kg of bw SPD, suggesting that SPD may protect DNA even when associated with SU consumption. However, the presence of sucrose appears to slightly reduce this effect, although not significantly.

Importantly, the comet assay performed in liver cells corroborated these findings and additionally demonstrated a mild genoprotective effect of SPD at the lower dose (10 mg/kg bw) but only in animals fed the SD. The comet assay is a rapid and sensitive technique for the quantitative assessment of DNA strand breaks based on the migration of denatured DNA fragments from the nucleus during electrophoresis. The extent of DNA migration from the comet head is directly proportional to the degree of damage induced by the genotoxic agent under evaluation.^49^

Regarding the safety of administration, SPD (maximum 60 mg/kg bw) did not cause experimental losses, significant post-mortem abnormalities, tissue degeneration, or relevant neoplasms.^50^ Prolonged supplementation with SPD (110 mg/kg bw) was shown to reduce cardiac neoplastic changes in both elderly and young mice,^11^ and attenuated inflammatory and oxidative processes (200 to 1000 μM) in macrophage and zebrafish models^51^ and protected rodents from hepatic endothelial injury when supplemented in drinking water at 3 mM.^52^ Gobert et al. demonstrated that the supplementation of rats with SPD (10 mg bw—the same dose tested in this study) could be a rational and economical therapy for colitis and thus a chemopreventive strategy against the development of colitis-associated carcinomas and potentially colorectal cancer.^53^ Furthermore, the interaction of SPD with DNA methylation, strongly reported in the literature,^54–59^ might explain the reduction of observed damage in experimental models, potentially influencing epigenetic regulation and protecting DNA.

In this work, we associated SPD with MMS in order to verify whether SPD had a protective effect against damage induced by MMS and whether the results were positive. MMS primarily damages DNA by adding methyl groups to specific sites on nucleotides such as guanine and adenine. This methylation can disrupt the DNA’s structure, leading to lesions that hinder replication and genetic stability. Additionally, MMS may exert secondary effects, such as altering the lipid composition in the nuclear membrane or modifying proteins through oxidation or acetylation. These indirect actions can influence DNA methylation regulation and other cellular mechanisms tied to genetic material integrity.^60^ Polyamines are known to modulate chromatin organization and essential cellular processes, implicating a significant role in genetic regulation.^61^ These findings support the hypothesis that SPD, through the modulation of DNA methylation and other epigenetic mechanisms, may contribute to maintaining genomic integrity and cellular function. SPD reduces DNA damage, likely by activating DNA repair and reducing the formation of ROS.^62^

Our results demonstrate that both dosages (10 and 30 mg/kg of bw) had antigenotoxic effects, with the 30 mg/kg of bw dose being more effective.

### 3.5 SOD and CAT Activities

The activities of the endogenous antioxidant defense enzymes, SOD and CAT, were quantified in hepatic cells of rodents (Figure 7A,B). In the CAT levels, the SD + SPD30 + MMS and SDSU + SPD30 + MMS groups showed a significant increase compared to those of the SD and SD + MMS groups. Additionally, the SD + SPD10 + MMS group also demonstrated higher levels compared to those of the SD + MMS group. The SDSU + MMS and SDSU + SPD10 + MMS groups showed a significant difference compared with the SD + SPD30 + MMS group. Finally, the SDSU + SPD30 + MMS group exhibited significantly increased levels compared to the SDSU + MMS and SD + SPD10 + MMS groups (Figure 7A). Regarding SOD, the SDSU + SPD10 + MMS group exhibited significantly higher SOD levels compared to the SD + MMS, SD + SPD10 + MMS, SD + SPD30 + MMS, and SDSU + MMS groups (Figure 7B).

**Figure 7:**
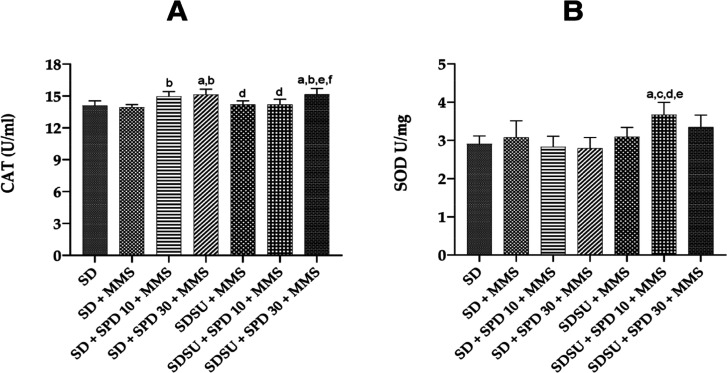
(A) CAT and (B) SOD quantification in liver cells of Swiss mice. CAT—catalase, SOD—superoxide dismutase, SD—SD Irradiated Nuvilab CR-1 (NC), MMS—methylmethanesulfonate 40 mg/kg body weight (bw) (PC), SU—sucrose, SPD 10—spermidine 10 mg/kg bw, and SPD 30—spermidine 30 mg/kg bw. Values are mean ± standard deviation. The animals received different treatments for 45 days, and on the 44th day (51st experimentation day), the mutagenic agent methylmethanesulfonate (MMS 40 mg/kg bw) was administered. Significance level for *p* < 0.05 (one-way ANOVA and Tukey’s test, average *n* = 6 animals per group). aSignificantly different from the SD group; bsignificantly different from the SD + MMS group; csignificantly different from the SD + SPD 10 + MMS group; dsignificantly different from the SD + SPD 30 + MMS group; esignificantly different from the SDSU + MMS group; and fsignificantly different from the SDSU + SPD 10 + MMS group.

Overall, these results suggest that SPD may interact with epigenetic regulatory mechanisms, with this interaction appearing to be more pronounced at a dose of 30 mg/kg body weight. However, it has been reported in the literature that certain bioactive compounds, including SPD, may also act as indirect or exogenous antioxidants by participating in free radical scavenging, reducing oxidative damage to age-related proteins, and limiting excessive ROS production.^63^ Free radicals and reactive molecules, which are more prevalent in biological systems, originate from ROS and reactive nitrogen species. These species are generated during electron transfer reactions involving the loss or gain of electrons. Excessive ROS production disrupts redox homeostasis, leading to oxidative stress and damage to critical biomolecules, including DNA, proteins, and cellular membranes. Oxidative stress, defined as an imbalance between the production and elimination of reactive species, plays a central role in the pathogenesis of chronic diseases and aging.^63,64^

Considering that the antioxidant potential of SPD is virtually nonexistent according to the DPPH assays due to the chemical nature of this polyamine, we investigated the ability of SPD to modulate the endogenous antioxidant defense system by quantifying the levels of SOD and CAT in the liver of the rodents. Our results showed that SPD (10 and 30 mg/kg bw) was able to promote a modest increase in CAT levels in rodents fed with SD and SDSU, as well as SOD (only at the 30 mg/kg bw dose in rodents fed with SDSU), demonstrating a possible effect on the modulation of the endogenous antioxidant defense system. Jiang demonstrated that SPD (110 mg/kg bw) significantly increased the activities of SOD and CAT in ovarian tissue, protecting against hepatic endothelial injury, enhancing autophagy, and reducing intracellular ROS.^63^ Similar results were reported by Yan et al., who found increased SOD levels and reduced ROS in cardiac tissue and in neonatal rat cardiomyocyte cultures.^65^ These findings suggest that SPD actively participates in the elimination of free radicals, the reduction of oxidative damage, and the overproduction of ROS, which may, at least in part, explain the chemopreventive mechanism addressed in this study.

On the other hand, MMS, an alkylating agent, contributes to increased oxidative stress by inducing DNA damage through the methylation of nitrogenous bases, particularly guanine and adenine. This process leads to the formation of lesions such as 7-methylguanine and 3-methyladenine, which render DNA chemically unstable and promote the generation of abasic sites and strand breaks—events that are closely associated with the production of ROS. In addition, MMS can interact with intracellular transition metals, thereby facilitating Fenton-type reactions and the generation of highly reactive hydroxyl radicals (•OH), further exacerbating cellular damage. Taken together, these mechanisms highlight the relevance of antioxidant-based strategies in mitigating oxidative stress and underscore the importance of compounds such as SPD in attenuating the deleterious effects induced by alkylating agents like MMS.^60,66–68^ Therefore, considering both the mechanism of action of the DNA-damaging agent and the observed protective effects of SPD, it can be inferred that the genoprotective activity of SPD is, at least in part, associated with its ability to reduce methylation-induced and oxidative damage to genetic material. This protective effect is further supported by the modulation of oxidative stress biomarkers, such as SOD and CAT, quantified in the present study.

## 4 Conclusion

Based on the results obtained, this study demonstrates that SPD supplementation exerts dose- and tissue-dependent protective effects against DNA damage in rodents. Specifically, the 10 mg/kg bw dose was not effective in reducing the MNPCE frequency in bone marrow, indicating a lack of antigenotoxic activity in this tissue. However, comet assay analysis revealed that this dose was sufficient to attenuate DNA damage in liver cells when administered in the absence of a SU diet, highlighting a differential tissue response to SPD supplementation. In contrast, SPD administered at 30 mg/kg bw consistently exhibited significant antigenotoxic effects, particularly in reducing DNA lesions in both the bone marrow and liver. Notably, the presence of a SU diet attenuated this protective effect, likely due to enhanced glucose metabolism and the consequent overproduction of ROS, which may partially counteract the antioxidant capacity of SPD. Mechanistically, the observed chemopreventive effects are supported by the ability of SPD to modulate oxidative stress, including the enhancement of antioxidant defenses, such as CAT, thereby contributing to the reduction of oxidative DNA damage.

Collectively, these findings provide novel evidence that SPD supplementation can mitigate genotoxic damage induced under conditions of metabolic stress, with greater efficacy observed at higher doses and in the context of a balanced diet. Nevertheless, the present study is limited by its experimental animal model and the restricted set of biomarkers evaluated. Therefore, further investigations involving additional molecular targets, longer exposure periods, and translational models are warranted to better elucidate the mechanisms underlying SPD-mediated chemoprotection and to assess its potential applicability in preventive strategies against diet-related genomic instability.

## Supplementary Material



## Notes

Nutritional information on SD; initial blood glucose, final blood glucose, and final minus initial blood glucose; and relative weight of heart, lungs, kidneys, spleen, gastrointestinal tract, and carcass (PDF)
