# Supplementary material for: Spermidine Supplementation Reduces Genetic Damage in the Liver and Bone Marrow of Rodents
Source: ACS Omega. 2026 Mar 10;11(11):17530–42. doi: 10.1021/acsomega.5c11099 (PMC13019200; doi:10.1021/acsomega.5c11099)
Supplement: Supplementary file 1 [file ao5c11099_si_001.pdf]

Spermidine Supplementation Reduces Genetic Damage in the Liver and Bone Marrow of Rodents

Janine Barcelos Chacon[1,2], Maria Clara Duarte[2], Michele Oliveira Carvalho[2], Patrícia Félix Ávila[1], Isabella Caroline Menon[2], Giulia de Mello Franco[2], Bruno Martins Dala Paula[1,3], Pollyanna Francielli de Oliveira[1,2*].

[1] Programa de Pós-graduação em Nutrição e Longevidade, Universidade Federal de Alfenas, Alfenas, UNIFAL, Rua Gabriel Monteiro da Silva, 700, Centro, Alfenas, MG, 37130-001, Brasil;
[2] Laboratório de Genética Humana, Instituto de Ciências da Natureza, Universidade Federal de Alfenas - UNIFAL, Rua Gabriel Monteiro da Silva, 700, Centro, Alfenas, MG, 37130-001, Brasil;
[3] Laboratório de Nutrição Experimental, Faculdade de Nutrição, Universidade Federal de Alfenas - UNIFAL, Rua Gabriel Monteiro da Silva, 700, Centro, Alfenas, MG, 37130-001, Brasil.

*Corresponding author:
Pollyanna Francielli de Oliveira, Universidade Federal de Alfenas, R. Gabriel Monteiro da Silva, 700 - Centro, Alfenas - MG, 37130-001, (+55) 35 99229-1701, (+55) 35 3701-9772, orcid.org/0000-0002-1646-0277 pollyanna.oliveira@unifal-mg.edu.br.

Supplementary material

S1. Nutritional information of SD.

**Macronutrients and micronutrients /100g**	**Amount**	TCV
Energy (kcal)	336 kcal	100%
Protein	22 g	26.19%
Fat	4 g	10.71%
Carbohydrates	53 g	63.09%
Crude Fiber	7 g	..
Calcium	1 – 1.4 g	..
Phosphorus	600 mg	..
Vitamin A	1300 mg	..
Vitamin D3	200 mg	..
Vitamin E	3.4 IU	..
Vitamin K3	0.3 mg	..
Vitamin B1	0.5 mg	..
Vitamin B2	0.6 mg	..
Vitamin B6	0.7 mg	..
Vitamin B12	2.2 mcg	..
Niacin	6 mg	..
Biotin	0.005 mg	..
Pantothenic Acid	2.1 mg	..
Choline	190 mg	..
Sodium	270 mg	..
Iron	5 mg	..
Manganese	6 mg	..
Zinc	6 mg	..
Copper	1 mg	..
Iodine	0.2 mg	..
Selenium	0.005 mg	..
Cobalt	0.15 mg	..
Fluorine	6 mg	..
Lysine	1.2 g	..
Methionine	400 mg	..

Basic components: whole corn, soybean meal, wheat bran. SD – Standard diet Irradiated Nuvilab CR-1, TCV - total caloric value, Kcal - kilocalorie, g - grams, mg - milligrams, IU - international unit, mcg - micrograms.

Source: manufacturer Nuvilab CR-1 Irratiated.

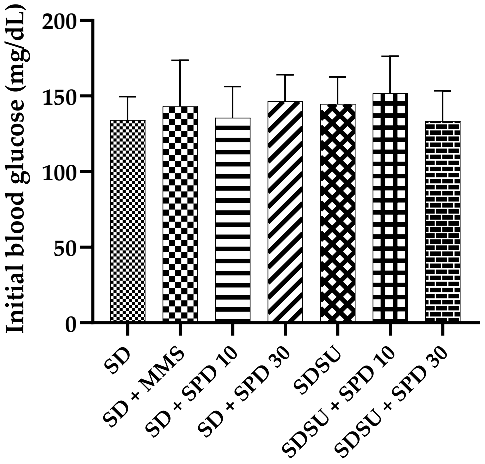

(a)

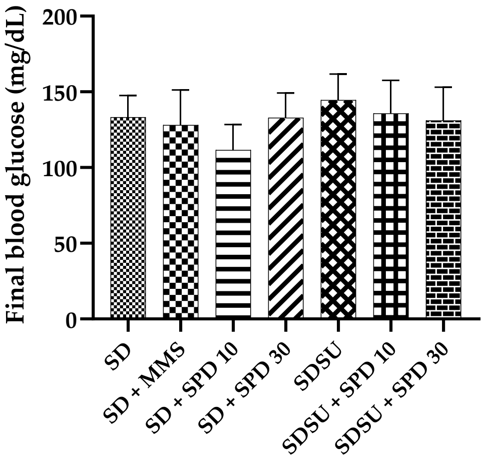

(b)

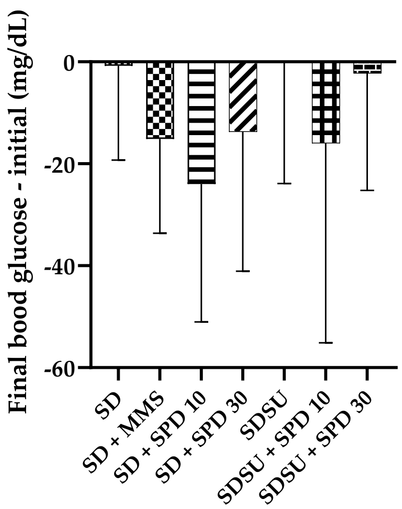

(c)

S2. (a) Initial blood glucose (mg/dL), (b) Final blood glucose (mg/dL), (c) Final blood glucose - initial (mg/dL). SD – Standard diet Irradiated Nuvilab CR-1 (negative control), MMS – methyl methanesulfonate 40 mg/kg body weight (bw) (positive control), SU – sucrose, SPD 10 – spermidine 10 mg/kg bw, SPD 30 – spermidine 30 mg/kg bw. Values are mean ± standard deviation. Significance level for $p<0.05$ (one-way ANOVA and Tukey's test, average n = 7 animals per group).

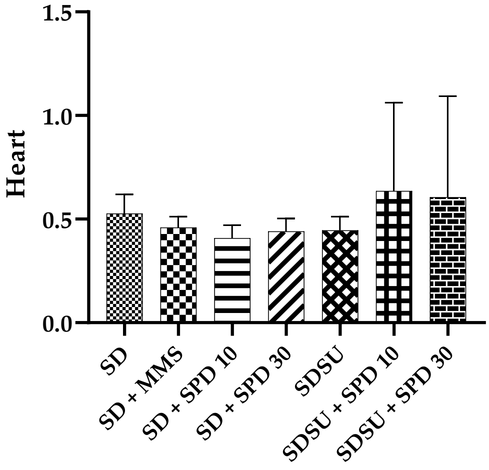

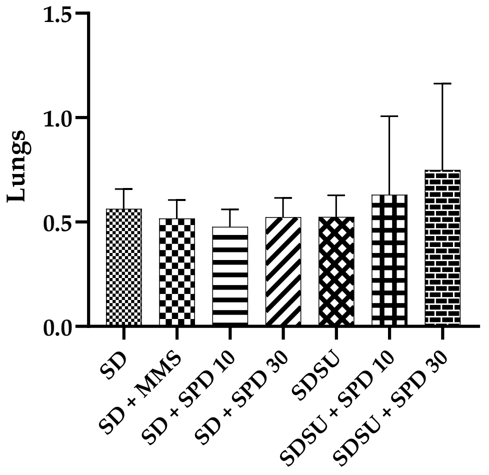

(a) (b)

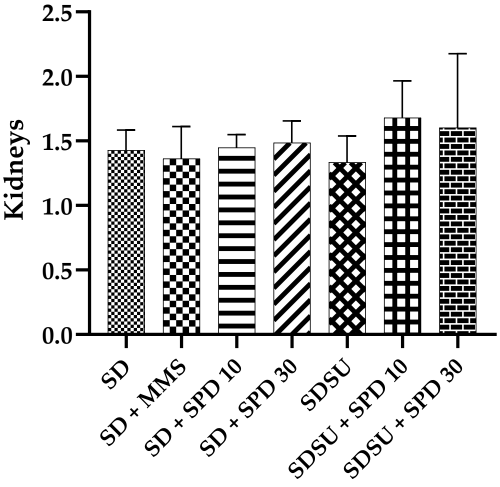

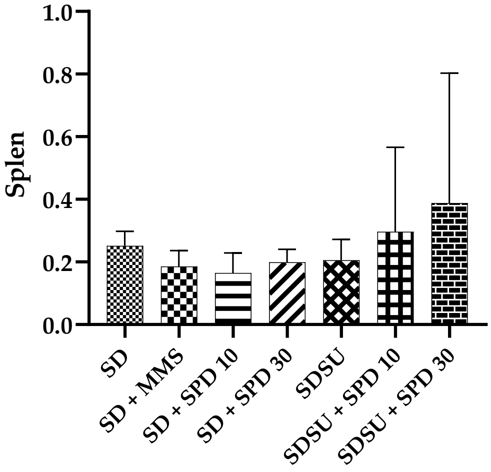

(c) (d)

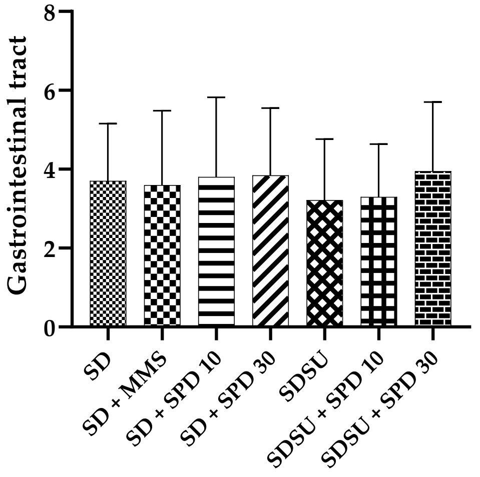

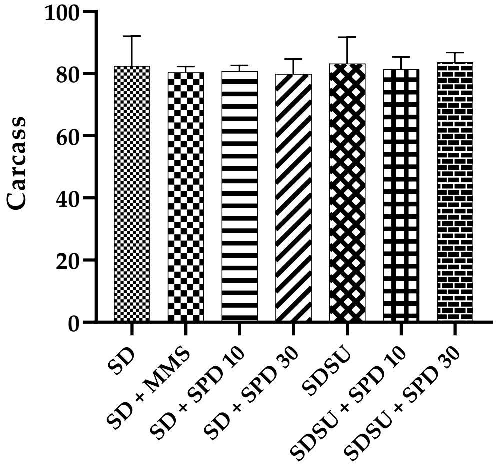

(e) (f)

S3. Relative weight of (a) Heart, (b) Lungs, (c) Kidneys, (d) Spleen, (e) Gastrointestinal tract, (f) Carcass. SD – Standard diet Irradiated Nuvilab CR-1 (negative control), MMS – methyl

methanesulfonate 40 mg/kg body weight (bw) (positive control), SU – sucrose, SPD 10 – spermidine 10 mg/kg bw, SPD 30 – spermidine 30 mg/kg bw. The values were calculated as relative weight (organ weight relative to body weight) and expressed as mean ± standard deviation. Significance level for $p<0.05$ (one-way ANOVA and Tukey's test, average n = 11 animals per group).
